# A pooled analysis of temporal trends in the prevalence of anxiety-induced sleep loss among adolescents aged 12–15 years across 29 countries

**DOI:** 10.3389/fpsyt.2023.1259442

**Published:** 2023-10-04

**Authors:** Guodong Xu, Lian Li, Lijuan Yi, Tao Li, Qiongxia Chai, Junyang Zhu

**Affiliations:** ^1^Ningbo Medical Center Lihuili Hospital, Zhejiang, China; ^2^Department of Psychiatry, Ningbo Kangning Hospital & Affiliated Mental Health Centre, Ningbo University, Zhejiang, China

**Keywords:** adolescents, prevalence, trends, anxiety-induced sleep loss, difference

## Abstract

**Background:**

Previous studies examining trends in sleep loss among adolescents have mainly focused on single countriy and region. This study aims to analyze temporal trends in the prevalence of anxiety-induced sleep loss among adolescents from 29 countries in five regions.

**Methods:**

This study used data from the Global School-based Student Health Survey 2003–2018, which surveyed 215,380 adolescents from 29 countries with at least two cross-sectional surveys per country. The weighted country-specific prevalence of anxiety-induced sleep loss and trends across the survey years were evaluated. Random- or fixed-effects meta-analyses were used to calculate pooled prevalence and temporal trends across 29 countries.

**Results:**

Temporal variations in anxiety-induced sleep loss across countries were identified. Increasing (Suriname, Vanuatu, and Myanmar), decreasing (Namibia, Jamaica, the Philippines, Samoa, and Indonesia), and stable (all other countries) trends in anxiety-induced sleep loss were noted. The pooled weighted prevalence of anxiety-induced sleep loss was 11.35 and 10.67% in the first and last surveys, respectively. There was no meaningful change in the propensity to have anxiety-related sleep disorders over time, with the reduction and OR of these two surveys being 0.54 (−0.53–1.61) and 0.98 (0.88–1.10). For subgroup analyses, no significant differences in pooled anxiety-induced sleep loss trends were seen between the two surveys for different sexes, regions, incomes, survey years in the first wave, survey periods, or number of surveys.

**Conclusion:**

Trends in the prevalence of anxiety-induced sleep loss in adolescents varied significantly across different countries. Generally, a stable trend was observed in 21 of the 29 countries surveyed. Our study provides data that can aid policymakers in establishing country-specific strategies for reducing anxiety-induced sleep loss in adolescents.

## 1. Introduction

Sleep is extremely important to health, especially for adolescents, who are in a formative period of development, with both their brains and bodies experiencing rapid growth. For adolescents, sufficient and good-quality sleep plays a vital role in mental, physical, and emotional development (1). Unfortunately, research indicates that many adolescents have poor quality and insufficient sleep (2), which can harm their physical growth. Anxiety has been consistently cited as one of the main causes of sleep loss (3), which can manifest as trouble in falling asleep, staying asleep, or waking up too early. Harvey’s cognitive model of insomnia supports this, as excessively negative cognitive activities, such as anxiety, are strongly associated with the initiation and persistence of sleep loss (4). Indeed, large studies have reported that anxiety is positively associated with sleep problems in both adolescents (5) and adults (6).

A population-based study of 82 countries reported the prevalence of anxiety-induced sleep loss to be 10% in adolescents aged 12–17 years (7). A 10-year time-trend analysis in Brazil showed that the prevalence of adolescents’ lost sleep from worry increased from 9.4% in 2006 to 9.6% in 2011 and then significantly increased to 13.3% in 2016 (8). The prevalence of daily difficulties falling asleep in Denmark adolescents increased from 7.0 to 13.4% in 1991–2018 (9). In addition, a large international study reported that 28 of 33 (84.8%) European countries/regions surveyed had an increasing prevalence of sleeping difficulties in adolescents (10). A decreasing trend in the prevalence of anxiety-induced sleep loss was reported in the Philippines from 2003 to 2011 (11) and in Lebanon from 2005 to 2017 (12). Besides, stable trends were noted from 2005/2006 to 2016 in the United Arab Emirates (12) and Morocco (13). A study in Korea suggested that the prevalence of individual meeting recommendations sleep guidelines were stable, and from 9.7% in 2013 to 10.3% in 2018 (14). However, most of these studies focused on single country or region, with pooled trends in the prevalence of adolescents’ anxiety-induced sleep loss in global being rarely studied.

Therefore, the aim of this study was to describe the trends in the prevalence of anxiety-induced sleep loss among adolescents using nationally representative datasets from 29 countries, and then examine the pooled trends across them. The findings of this study provide invaluable evidence for the development and implementation of relevant policies and programs in worldwide to address this issue.

## 2. Methods

The Global School-based Student Health Survey (GSHS) is a multi-country, repeated, cross-sectional survey that was conducted by the World Health Organization and the US Centers for Disease and Control and Prevention. The core aim of this survey was to investigate the prevalence of, and trends in, healthy and unhealthy behaviors and major noncommunicable diseases among school-aged adolescents worldwide. The multi-survey data were obtained from the GSHS. The specific questionnaires used in the survey are available on the WHO^1^ and CDC^2^ websites. Briefly, 12–15-year-old adolescents in education were selected using two-stage cluster sampling for each survey (15). In the first stage of the cluster sampling, schools were randomly selected from a list of all schools in the country using the probability proportionate to size method. In the second stage of the sampling process, several classrooms that included high proportions of students of the target age were selected for inclusion from within each of the participating schools, and all students in the selected classes were included in the sampling frame. To allow direct comparisons to be made between each country, the standardized questionnaire modules used were those for which test–retest reliability had already been established (16) and that had been translated into the appropriate languages with no changes in the questions. All data collection was performed during regular class time using computer-scannable sheets. All surveys conducted as part of the GSHS worldwide were approved by the ministry of health or education and an institutional review board or ethics committee in each country. Adolescents voluntarily took part in the GSHS, and verbal or written consent was obtained from each adolescent and their parents or guardians.

From the publicly available GSHS data, we identified countries that participated in at least two nationally representative surveys from 2003 to 2018 and whose datasets included anxiety-induced sleep loss as a variable.

Ultimately, data from 29 countries were included in our study (Supplementary Figure S1). Information relating to anxiety-induced sleep loss was obtained with the question “During the past 12 months, how often have you been so worried about something that you could not sleep at night?” (13) with response options of 1 (never), 2 (rarely), 3 (sometimes), 4 (most of the time), and 5 (always) with scores coded as 1–3 = 0 and 4–5 = 1, The adolescents who were identified as having anxiety-induced sleep loss were coded as 1. Confounding variables included sex, age, and hunger. Hunger was evaluated with the question “How often did you go hungry because there was not enough food in your home during the past 30 days?” with the same response options of 1 (never) to 5 (always).

### 2.1. Statistical analysis

The prevalence of anxiety-induced sleep loss was reported by weighted prevalence and 95% confidence intervals (CIs) using the SAS software PROC SURVEYMEANS procedure. We added weights, strata, and a primary sampling unit to every set of school-aged adolescents to reflect the weighting process and the two-stage sampling design. The pooled estimates of prevalence and the differences between countries were calculated by fixed−/random-effects meta-analysis using STATA and the Higgins’s I^2^ statistic was used to estimate between-country heterogeneity. An I^2^ value <25% was considered as negligible heterogeneity and a fixed-effects meta-analysis was performed. Otherwise, we used a random-effects meta-analysis for analysis. After adjusting for sex, age and hunger, the between-survey differences and country-specific estimated odds ratio (OR) for the prevalence of anxiety-induced sleep loss were estimated using the SAS software PROC SURVEYREG and PROC SURVEYLOGISTIC procedure (17, 18). Subgroup analyses were stratified by sex (males vs. females), WHO region [Africa (AFR), Americas (AMR), Eastern Mediterranean (EMR), Western Pacific (WPR) and South-East Asia (SEAR)], World Bank income level (low, lower-middle, upper-middle and high), survey year in first wave (<2008 and ≥ 2008), inter-survey period (≤ 7 years and > 7 years) and number of surveys (2 and > 2). Sensitivity analyses were conducted to determine the stability of the pooled estimates after excluding each study in turn. Meta-regression analysis was used to explore the sources of heterogeneity. SAS version 9.4 (SAS Institute, Cary, NC, United States) and STATA version 12.0 (Stata Corporation, College Station, TX) were used to perform statistical analyses. A two-sided *p* values less than 0.05 were considered statistically significant.

## 3. Results

Of the countries included in this study, three were in AFR, ten were in AMR, four were in EMR, seven were in WPR and five were in SEAR (Table 1). Three countries were low-income, eight were lower middle-income, eleven were upper middle-income and seven were high income. The initial survey in each country was conducted from 2003 to 2011, and the last surveys were conducted from 2010 to 2018. The median inter-survey period was 7 years. The response rates ranged from 96.76% in Kuwait (2015) to 100% in Benin and the Cook Islands (2009) (Table 1).

**Table 1:** Survey characteristics.

	**Region**	**Income**	**Survey year**	**Response rate %**	**Sample size**^**a**^	**Boys %**
Benin	AFR	L	2009	100	1,170	63.31
			2016	99.30	712	44.98
Namibia	AFR	UM	2004	98.45	4,459	44.06
			2013	99.12	1919	41.46
Seychelles	AFR	H	2007	98.87	1,141	46.87
			2015	98.45	2029	46.75
Kuwait	EMR	H	2011	98.13	2,255	47.98
			2015	96.76	1968	45.55
Lebanon	EMR	UM	2005	99.07	4,482	45.13
			2011	99.70	1976	46.33
			2017	99.10	3,317	40.92
Morocco	EMR	LM	2006	98.64	1959	47.65
			2010	98.00	2,357	50.00
			2016	98.79	3,927	50.22
United Arab Emirates	EMR	H	2005	98.95	12,684	47.36
			2010	98.87	2,276	38.78
			2016	98.59	3,422	47.18
Anguilla	AMR	L	2009	99.00	694	47.90
			2016	97.87	552	48.45
Argentina	AMR	H	2007	99.61	1,531	47.13
			2012	99.34	21,386	46.79
			2018	99.27	36,211	47.78
Guatemala	AMR	L	2009	99.51	4,495	44.98
			2015	96.65	3,490	48.14
Guyana	AMR	UM	2004	98.04	1,049	40.10
			2010	99.29	1959	44.60
Jamaica	AMR	UM	2010	99.58	1,199	48.87
			2017	98.59	1,046	45.11
Saint Lucia	AMR	UM	2007	99.72	1,069	42.08
			2018	97.69	1,310	44.04
Saint Vincent and the Grenadines	AMR	UM	2007	99.24	1,179	45.75
			2018	97.76	1,005	45.76
Suriname	AMR	UM	2009	99.81	1,044	46.78
			2016	99.72	1,449	44.84
Trinidad and Tobago	AMR	H	2007	99.35	2,434	47.89
			2011	99.45	2,350	54.79
			2017	99.49	2,749	46.56
Uruguay	AMR	H	2006	99.93	2,880	45.67
			2012	98.50	2,826	47.00
Cook Islands	WPR	H	2011	99.53	845	49.11
			2015	100	366	48.48
Fiji	WPR	UM	2010	99.80	1,492	42.00
			2016	98.63	1,516	49.23
Mongolia	WPR	LM	2010	99.85	3,323	43.67
			2013	99.57	3,691	47.63
Philippines	WPR	LM	2003	99.64	4,183	39.72
			2007	97.36	3,392	40.35
			2011	99.79	3,837	41.11
			2015	99.89	6,155	43.57
Samoa	WPR	UM	2011	96.09	2,114	39.99
			2017	99.24	1,050	33.30
Tonga	WPR	UM	2010	99.23	1931	44.75
			2017	98.74	2041	46.90
Vanuatu	WPR	LM	2011	100	852	41.71
			2016	99.61	1,283	40.38
Maldives	SEAR	LM	2009	98.69	1955	43.34
			2014	97.92	1744	40.72
Myanmar	SEAR	LM	2007	99.42	2,214	48.06
			2016	99.11	2,217	45.80
Indonesia	SEAR	LM	2007	99.50	3,007	47.15
			2015	99.63	8,773	46.19
Sri Lanka	SEAR	LM	2008	99.64	2,495	45.53
			2016	98.85	2,228	43.15
Thailand	SEAR	UM	2008	99.59	2,664	48.83
			2015	98.06	4,052	46.53

AFR, African Region; EMR, Eastern Mediterranean Region; AMR, Region of the Americas; WPR, Western Pacific Region; SEAR, South-East Asia Region; L, low income; LM, lower middle-income; UM, upper middle-income; H, high income. Income classification is based on the World Bank classification at the time of the survey.

^a^Based on sample aged 12–15 years.

**Figure 1:**
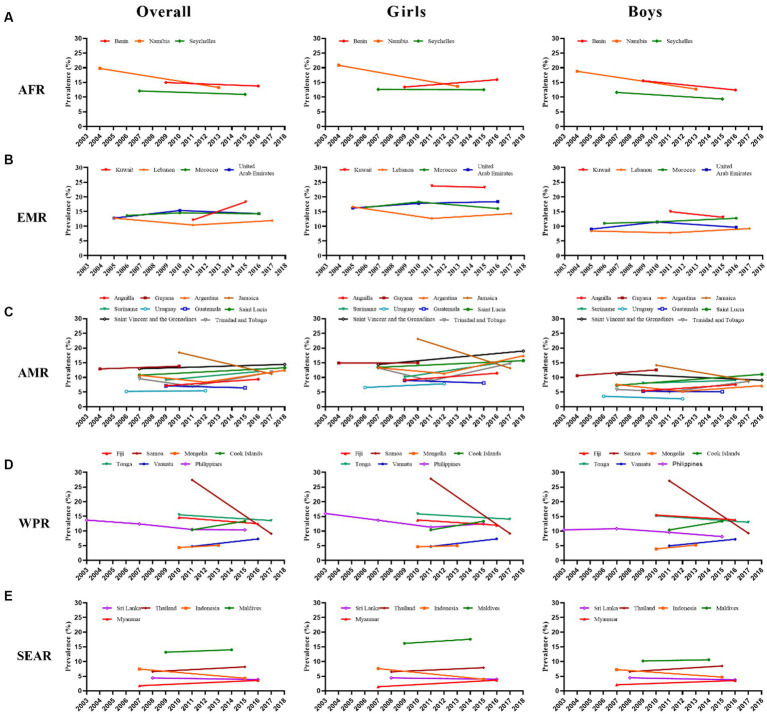
Trends in prevalence (%) of anxiety-induced sleep loss by country, region, and sex. **(A)** African Region (AFR), **(B)** Eastern Mediterranean Region (EMR), **(C)** Region of the Americas (AMR), **(D)** Western Pacific Region (WPR), **(E)** South-East Asia Region (SEAR).

**Figure 2:**
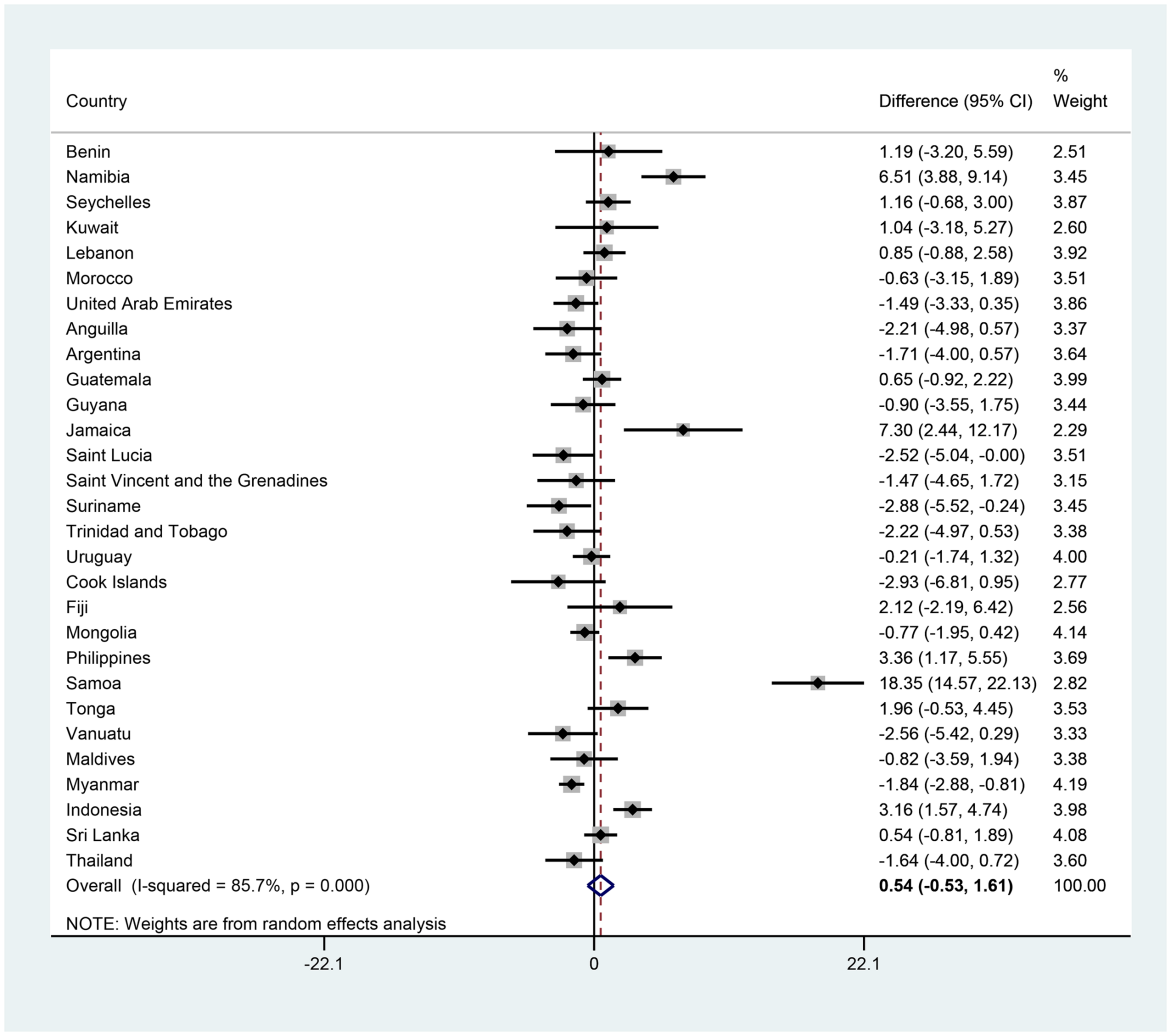
The country-specific and pooled difference of anxiety-induced sleep loss. The black diamonds and horizontal line represent the difference and their 95% confidence interval (CI), the gray box size represents the weight of the country, and the bottom diamond represents the pooled difference and 95% CI.

The country-specific and pooled estimates of anxiety-induced sleep loss prevalence, and their trends, are shown in Figure 1 and Supplementary Table S1. In the initial survey, the prevalence of anxiety-induced sleep loss ranged from 1.75% (1.05–2.45%) in Myanmar to 27.44% (23.87–31.02%) in Samoa (Supplementary Table S1 and Figure 1). On a regional scale, prevalence ranged from 6.56% (3.54–9.58%) in SEAR to 15.58% (10.47–20.69%) in AFR (Table 2). In the last survey, the prevalence of anxiety-induced sleep loss ranged from 3.59% (2.79–4.39%) in Myanmar to 18.31% (14.80–21.82%) in Kuwait (Supplementary Table S1 and Figure 1) and ranged from 10.03% (7.45–12.62%) in WPR to 14.20% (12.28–16.11%) in EMR (Table 2). The reduction in the prevalence of anxiety-induced sleep loss between the first and last surveys ranged from −2.88% (−5.52−−0.24%) in Suriname to 18.35% (14.57–22.13%) in Samoa (Figure 2 and Supplementary Table S1).

**Table 2:** Subgroup prevalence and trend of anxiety based on 29 countries from the global school-based student health surveys.

	Prevalence on first survey, %	Prevalence on last survey, %	Difference (95%CI)	OR (95%CI)
All countries (*n* = 29)	11.35 (9.57–13.12)	10.67 (9.13–12.21)	0.54 (−0.53–1.61)	0.98 (0.88–1.10)
WHO region				
AFR (*n* = 3)	15.58 (10.47–20.69)	13.37 (10.52–14.23)	3.04 (−0.77–6.84)	0.81 (0.61–1.09)
AMR (*n* = 10)	9.97 (8.20–11.74)	10.99 (8.77–13.21)	−0.95 (−2.15–0.25)	1.09 (0.97–1.23)
EMR (*n* = 4)	14.40 (12.10–16.70)	14.20 (12.28–16.11)	−0.23 (−1.50–1.03)	1.02 (0.92–1.13)
WPR (*n* = 7)	12.87 (7.60–18.14)	10.03 (7.45–12.62)	2.69 (−1.31–6.69)	0.87 (0.61–1.28)
SEAR (*n* = 5)	6.56 (3.54–9.58)	10.67 (9.13–12.21)	−0.07 (−2.06–1.92)	1.07 (0.73–1.56)
Income				
H (*n* = 7)	11.36 (8.85–13.86)	12.18 (9.23–15.14)	−0.74 (−1.81–0.32)	1.07 (0.84–1.39)
UM (*n* = 11)	14.44 (11.49–17.38)	12.10 (10.99–13.22)	2.35 (−0.64–5.33)	0.86 (0.69–1.08)
LM (*n* = 8)	7.81 (5.09–10.53)	7.81 (5.11–10.50)	0.11 (−1.36–1.57)	0.92 (0.66–1.27)
L (*n* = 3)	9.59 (5.64–13.53)	9.60 (5.58–13.62)	−0.15 (−2.13–1.84)	1.04 (0.80–1.35)
Survey year in first wave				
<2008(*n* = 14)	11.12 (8.41–13.82)	11.54 (9.18–13.91)	0.44 (−0.86–1.74)	0.98 (0.85–1.12)
≥2008(*n* = 15)	10.77 (8.48–13.07)	10.57(8.43–12.71)	0.69 (−1.19–2.56)	0.99 (0.81–1.23)
Survey period				
≤7 years (*n* = 15)	12.02 (9.56–14.47)	10.78 (8.78–12.77)	1.08 (−0.80–2.95)	0.96 (0.79–1.16)
>7 years (*n* = 14)	10.35 (7.95–13.36)	10.54 (8.16–12.93)	0.15 (−1.13–1.42)	1.01 (0.87–1.16)
Number of surveys				
2 (*n* = 23)	11.13 (9.08–13.17)	10.17 (8.48–11.86)	0.79 (−0.50–2.09)	0.97 (0.83–1.12)
>2 (*n* = 6)	12.22 (11.00–13.44)	12.48 (11.33–13.62)	−0.24 (−1.89–1.41)	1.05 (0.91–1.21)

AFR, African Region; EMR, Eastern Mediterranean Region; AMR, Region of the Americas; WPR, Western Pacific Region; SEAR, South-East Asia Region; L, low income; LM, lower middle-income; UM, upper middle-income; H, high income. Income classification is based on the World Bank classification at the time of the survey.

**Figure 3:**
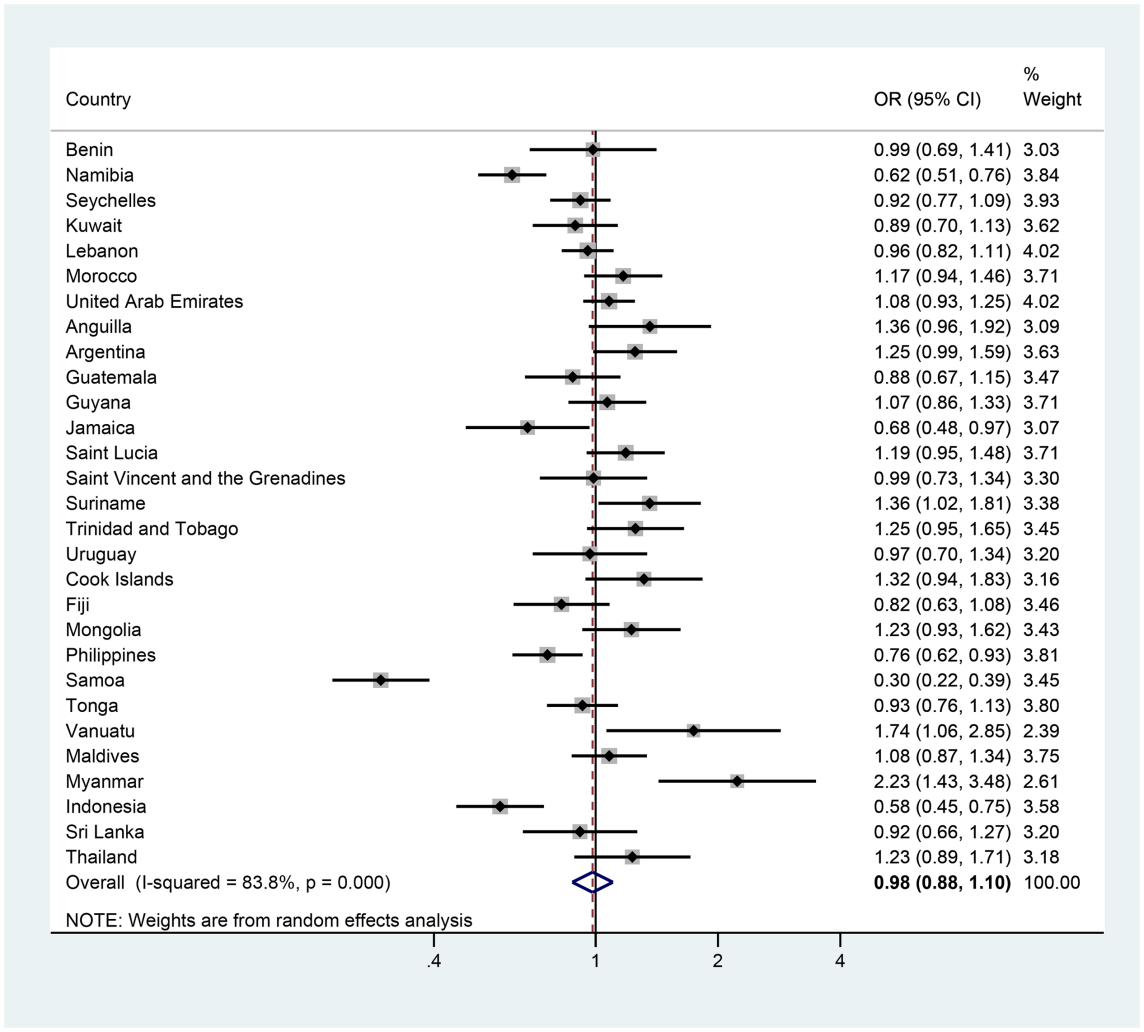
The country-specific and pooled estimated trend of anxiety-induced sleep loss. The black diamonds and horizontal line represent the OR and their 95% confidence interval (CI), the gray box size represents the weight of the country, and the bottom diamond represents the pooled OR and 95% CI.

The pooled estimates of anxiety-induced sleep loss prevalence were 11.35 and 10.67% in the first and the last survey, respectively; however, the adjusted reduction and adjusted OR (AOR) for the two surveys was 0.54 (−0.53–1.61) and 0.98 (0.88–1.10), suggesting that there was no significant trend in the prevalence of anxiety-induced sleep loss between the first and last surveys in adolescents (Figures 2, 3 and Supplementary Table S1). In terms of countries, five countries (Namibia, Jamaica, the Philippines, Samoa and Indonesia) exhibited significant decreases in the prevalence of anxiety-induced sleep loss over the study period, with AORs ranging from 0.30 in Samoa to 0.76 in the Philippines (Figure 3 and Supplementary Table S1). In contrast, the prevalence of anxiety-induced sleep loss significantly increased in three countries (Suriname, Vanuatu, and Myanmar), with AORs ranging from 1.36 in Suriname to 2.23 in Myanmar (Figure 3 and Supplementary Table S1). In the other 21 countries, the prevalence of anxiety-induced sleep loss remained stable over the study period (Figure 3 and Supplementary Table S1).

**Figure 4:**
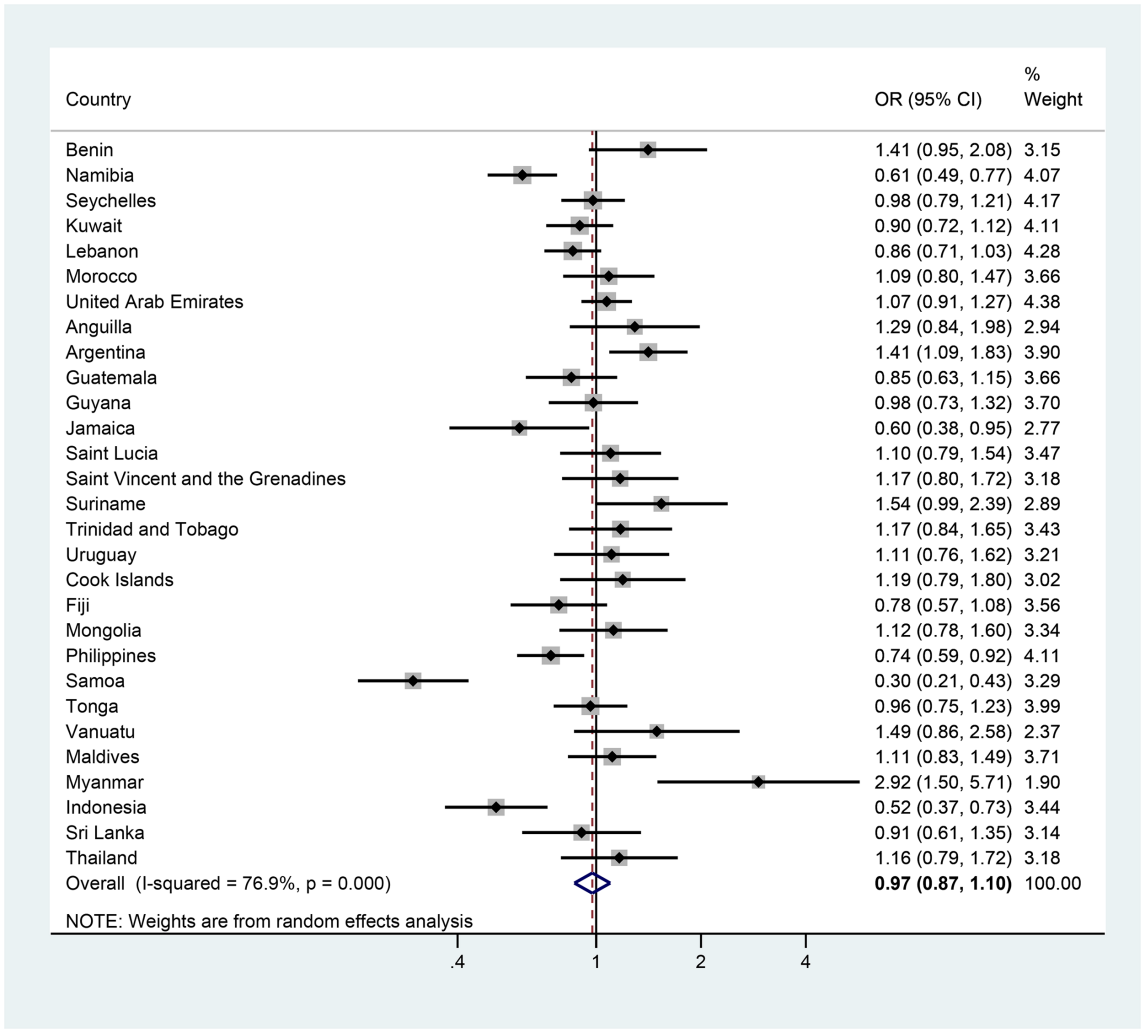
The country-specific and pooled estimated trend of anxiety-induced sleep loss in girls. The black diamonds and horizontal line represent the OR and their 95% confidence interval (CI), the gray box size represents the weight of the country, and the bottom diamond represents the pooled OR and 95% CI.

**Figure 5:**
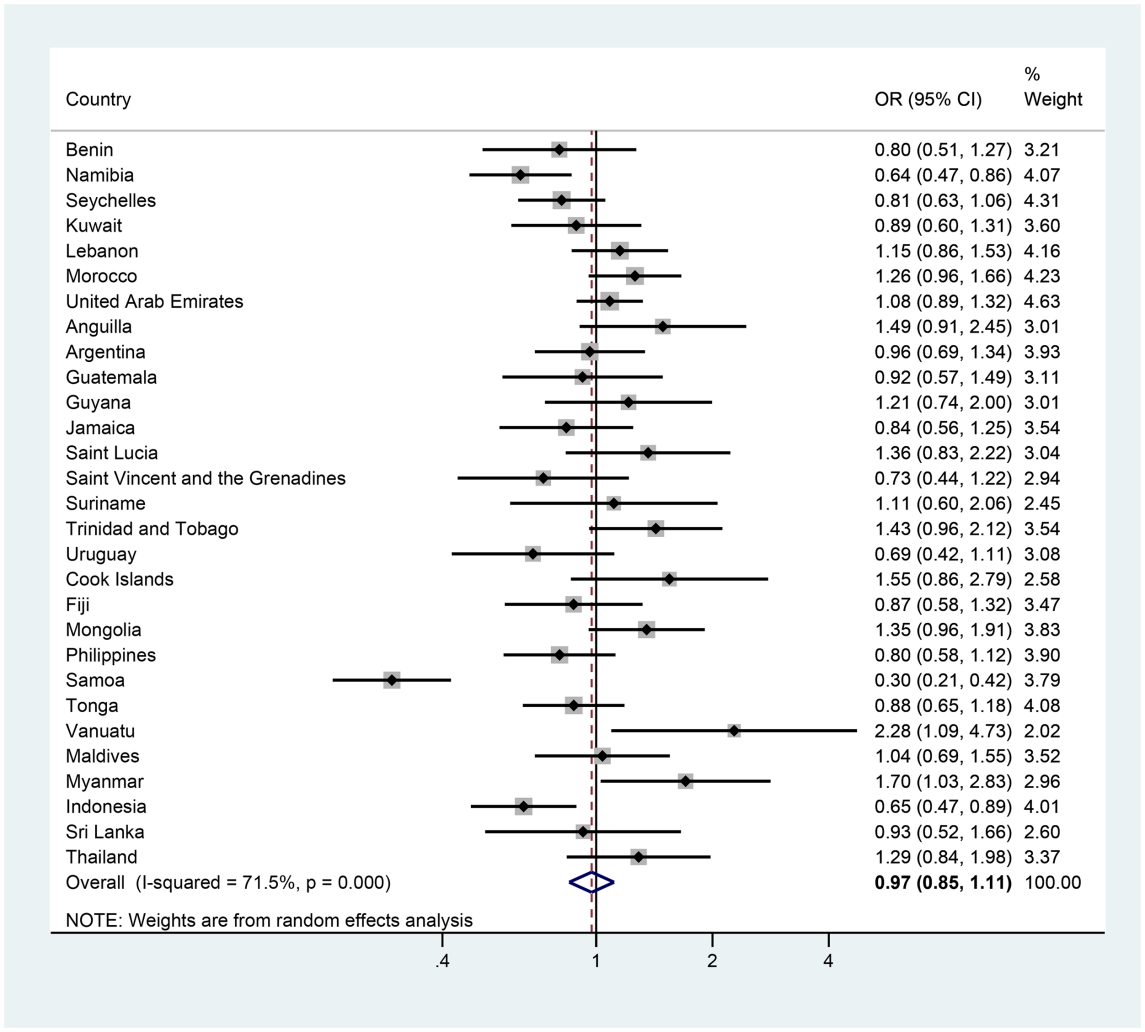
The country-specific and pooled estimated trend of anxiety-induced sleep loss in boys. The black diamonds and horizontal line represent the OR and their 95% confidence interval (CI), the gray box size represents the weight of the country, and the bottom diamond represents the pooled OR and 95% CI.

In girls specifically, 7 of the 29 countries demonstrated significant changes in the prevalence of anxiety-induced sleep loss between the first and last surveys. Five countries (Namibia, Jamaica, the Philippines, Samoa and Indonesia) exhibited a significant decrease in prevalence, with AORs was ranging from 0.30 in Samoa to 0.74 in the Philippines (Figure 4 and Supplementary Table S2). In contrast, two countries (Argentina and Myanmar) experienced a significant increase, with AORs of 1.41 and 2.92 (Figure 4 and Supplementary Table S2). The remaining 22 countries showed a stable trend in the prevalence of anxiety-induced sleep loss (Figure 4 and Supplementary Table S2). In boys, three countries (Namibia, Samoa and Indonesia) experienced significant decreases in the prevalence of anxiety-induced sleep loss, with AORs ranging from 0.30 in Samoa to 0.65 in Indonesia (Figure 5 and Supplementary Table S3). Conversely, two countries (Vanuatu and Myanmar) displayed significant increases in prevalence, with AORs of 2.28 and 1.70, respectively (Figure 5 and Supplementary Table S3). The remaining 24 countries had a stable trend (Figure 5 and Supplementary Table S3).

After adjusting for sex, age and hunger, no significant changes were identified in the prevalence of anxiety-induced sleep loss between the first and last surveys for different regions, income levels, survey year in first wave, lengths of survey period or number of surveys (Table 2). Sensitivity analyses did not alter any of these global trends in the prevalence of anxiety-induced sleep loss (Supplementary Figure S4) and the result of meta-regression also confirmed it (*p* = 0.902).

## 4. Discussion

This study found that the prevalence of anxiety-induced sleep loss varied widely across the 29 countries, with most countries having high prevalence of such anxiety-induced sleep loss. We also described plateauing, increasing, and decreasing trends in the prevalence of anxiety-induced sleep loss across different countries and regions.

A previous global study of 82 countries reported the prevalence of sleep loss over anxiety to be 10% in 12–17-year-old adolescents (7). Given that sleep loss significantly correlates with suicidal ideation, plans and attempts (19), it is extremely important to address sleep loss in children. In our study, the pooled weighted prevalence of anxiety-induced sleep loss was 11.35 and 10.67% in the first and last surveys and the OR of these two surveys was 0.98 (0.88–1.10), suggesting a plateauing trend in the prevalence of anxiety-induced sleep loss in adolescents in 29 countries. The high prevalence of anxiety-induced sleep loss might be due to environmental factors, social inequality (9) or policy (7). Based on the GSHS data, smoking (2), bullying and victimization (20), reduced fruit intake (21) and sedentary behavior (22) are all related to increased prevalence of anxiety-induced sleep loss in adolescents. Unfortunately, numerous studies have found that such behaviors have remained common in adolescents over time (23), with some even increasing in prevalence, such as sedentary behavior (24). In addition, a previous study conducted in GSHS reported that nearly one in three of these countries had no specific mental health policy (7), suggesting significant neglection of mental health and a shortage of appropriate health services in those countries.

We noted a consistently higher prevalence of anxiety-induced sleep loss in girls than in boys in Supplementary Tables S2, S3, which aligns with studies carried out in Canada (25), the United States (26) and Brazil (8). Indeed, sex inequality in the prevalence of anxiety-induced sleep loss has long been reported (26), suggesting that a tendency for severe anxiety in females has not abated. Females are more likely to be both anxious and stressed than males, potentially due to higher estrogen and progesterone levels in their bodies (27). Moreover, females tend to experience mistreatment more often and have been reported to internalize their distress more than their male counterparts (28). In fact, suicidal ideation and anxiety were both higher in females than their male counterparts (7). As such, more attention should be paid to the mental status of females. However, plateauing trends of the two surveys in the prevalence of anxiety-induced sleep loss were both shown in boys and girls, which further suggested the clinically meaningful of reducing anxiety-induced sleep loss. In addition, the prevalence is greater in higher income countries, which was consisted with the pervious study (2), however, in another study (7) conducted in GSHS showed low-income countries have the highest prevalence of anxiety-induced sleep loss, the difference may be due to varied country were included.

In our study, 6 of the 29 countries examined had conducted more than two surveys. However, only the Philippines exhibited a decreasing trend in the prevalence of anxiety-induced sleep loss. Such improvements might be due to the existence of school-based mental health education and other public health strategies in the Philippines (29, 30). Indeed, multiple surveys suggesting a stable high prevalence of anxiety-induced sleep loss should warrant the attention of state authorities in other countries. The findings of the GSHS have important implications for curbing the stable high prevalence of anxiety-induced sleep loss. Effective policies (e.g., rational emotive behavioral education and social emotional learning) should be implemented to mitigate anxiety-induced sleep loss in school-aged adolescents (31). Furthermore, healthy lifestyles that could effectively reduce anxiety in adolescents should be advocated (32). Parental and peer relationships have strong association with adolescent anxiety (7). Thus, parents and educators should maintain parental and peer relationships with adolescents. Additionally, adolescents should be taught coping mechanisms for dealing with sleep loss, such as going to bed at a consistent time and reducing the use of electronic devices, especially in the hour before bedtime (33). Finally, excessive use of electronic devices has been associated with both anxiety and insomnia (34, 35), especially when used at bedtime. It is worth noting that the long-term frequent use of electronic devices has become more prevalent in recent years in many countries (36), which may be contributing to insomnia.

This is the first study of global trends in the prevalence of anxiety-induced sleep loss in adolescents. Using GSHS data provides this study with the strength of standardized data collection and a regular data collection time. Moreover, the survey data collected for each country was nationally representative, due to a large random sample drawn from a wide variety of geographic and cultural settings. However, some limitations also need to be acknowledged. First, only 29 countries had at least two survey waves, which limits the generalizability of our findings to worldwide. Second, only school-attending adolescents aged 12–15 years were included in our study, and thus, selection bias may exist within the samples due to low school attendance in some countries. It is possible that the characteristics of school-attending adolescents changed over the study period, resulting in an additional influence on the temporal trends observed in our study. Therefore, future studies of temporal trends in the prevalence of anxiety-induced sleep loss should also include non-school-attending adolescents, to represent the entire adolescent population. Third, anxiety-induced sleep loss was measured using a single self-report item, which may have resulted in bias being introduced into this study. Fourth, the various surveys included in this study were conducted in different years and had different survey period. Therefore, the temporal trends identified are not comparable across countries. However, the results did not differ significantly stratified by the median of the year of the first survey (2008) or survey period (7 years). In addition, high heterogeneity was found across countries, and random-effects model ws used to reduce the effect. Finally, given that some countries provided more survey data, the accuracy of our findings may vary between countries.

In conclusion, our study reports that the trends in the prevalence of anxiety-induced sleep loss vary significantly across countries, with a stable prevalence of anxiety-induced sleep loss seen across 29 countries. These findings highlight the need to develop specific interventions to mitigate anxiety-induced sleep loss in adolescents. Our study provides data that policymakers can use to establish country-specific strategies for reducing anxiety-induced sleep loss in adolescent populations.

## Data availability statement

The original contributions presented in the study are included in the article/Supplementary material, further inquiries can be directed to the corresponding author.

## Ethics statement

The studies involving humans were approved by the all surveys conducted as part of the GSHS worldwide were approved by the ministry of health or education and an institutional review board or ethics committee in each country. Adolescents voluntarily took part in the GSHS, and verbal or written consent was obtained from each adolescent and their parents or guardians. The studies were conducted in accordance with the local legislation and institutional requirements. Written informed consent for participation was not required from the participants or the participants’ legal guardians/next of kin in accordance with the national legislation and institutional requirements.

## Author contributions

GX: Writing – original draft, Writing – review & editing. LL: Investigation, Methodology, Writing – original draft. LY: Investigation, Methodology, Writing – original draft. TL: Data curation, Writing – original draft. QC: Data curation, Writing – original draft. JZ: Writing – original draft, Writing–review–&–editing.

## Footnotes

^1^ http://www.who.int/chp/gshs/en

^2^ https://www.cdc.gov/gshs/index.htm
