# Supplementary material for: A pooled analysis of temporal trends in the prevalence of anxiety-induced sleep loss among adolescents aged 12–15 years across 29 countries
Source: Front Psychiatry. 2023 Oct 4;14:1259442. doi: 10.3389/fpsyt.2023.1259442 (PMC10582330; doi:10.3389/fpsyt.2023.1259442)
Supplement: Supplementary file 1 [file Data_Sheet_1.docx]

**
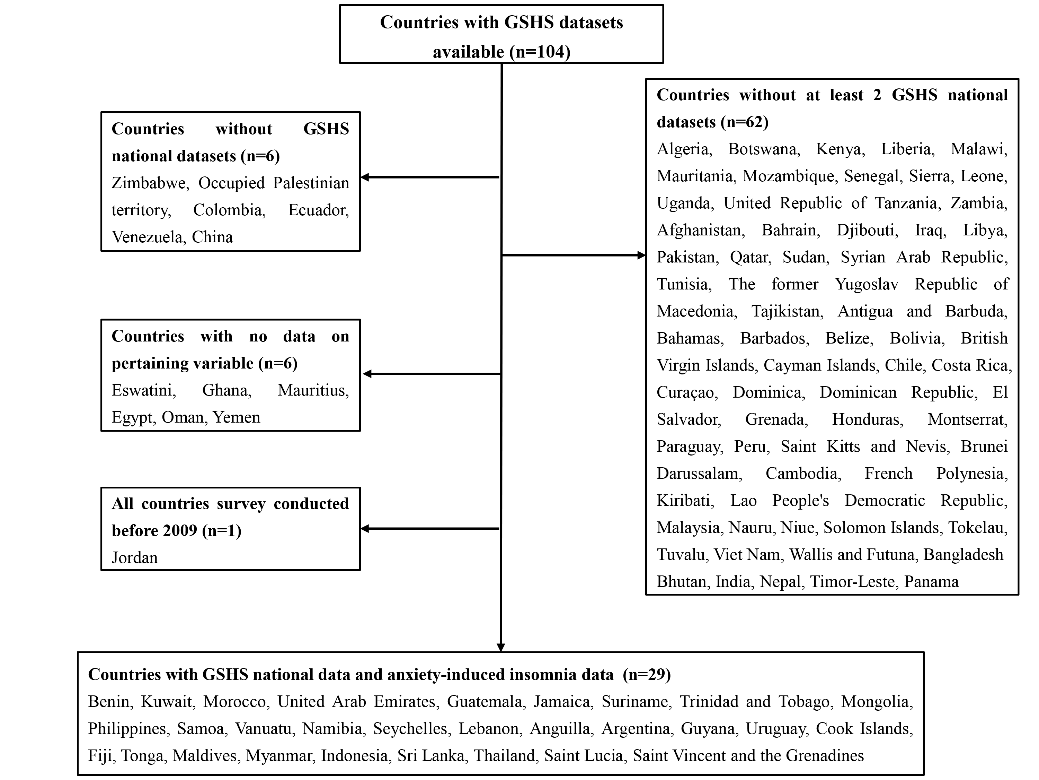
**

**SUPPLEMENTARY FIGURE 1**

Selection process for countries using GSHS national data.

**
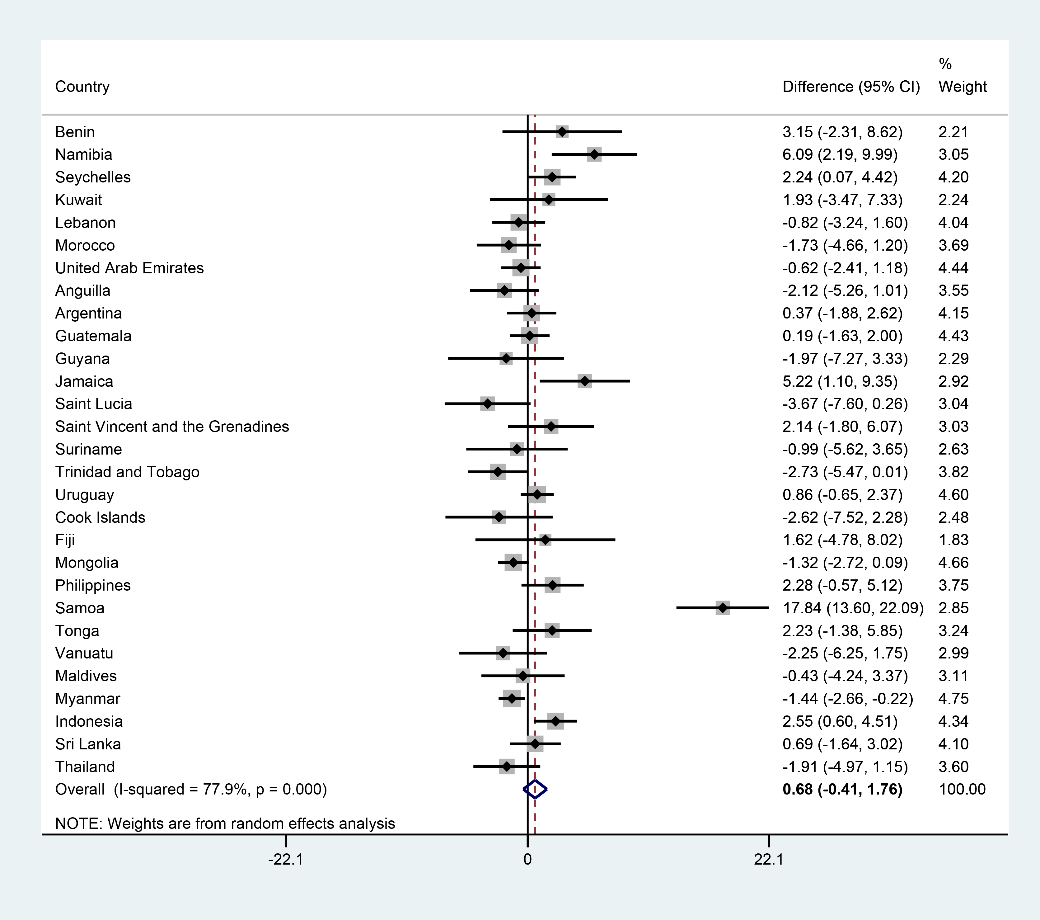
**

**SUPPLEMENTARY FIGURE 2**

The country-specific and pooled difference of anxiety in girls. The black diamonds and horizontal line represent the difference and their 95% confidence interval (CI), the gray box size represents the weight of the country, and the bottom diamond represents the pooled difference and 95% CI.

**
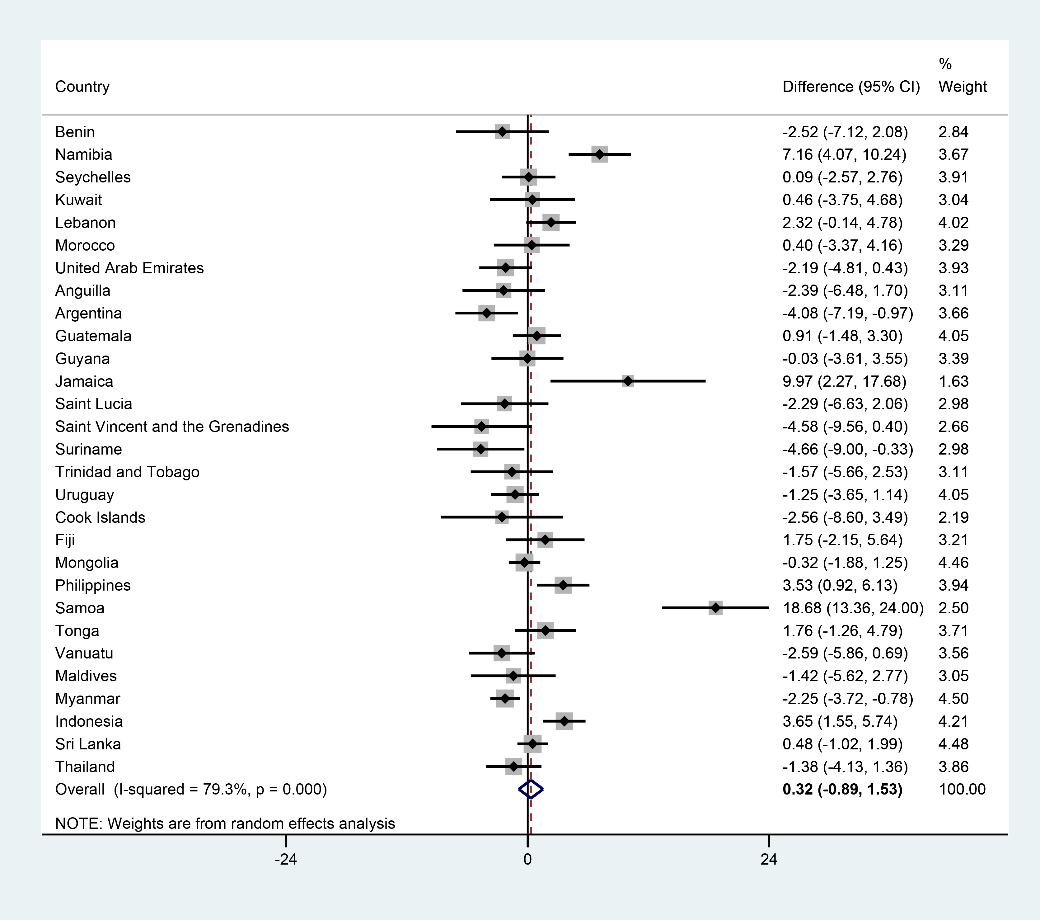
**

**SUPPLEMENTARY FIGURE 3**

The country-specific and pooled difference of anxiety in boys. The black diamonds and horizontal line represent the difference and their 95% confidence interval (CI), the gray box size represents the weight of the country, and the bottom diamond represents the pooled difference and 95% CI.


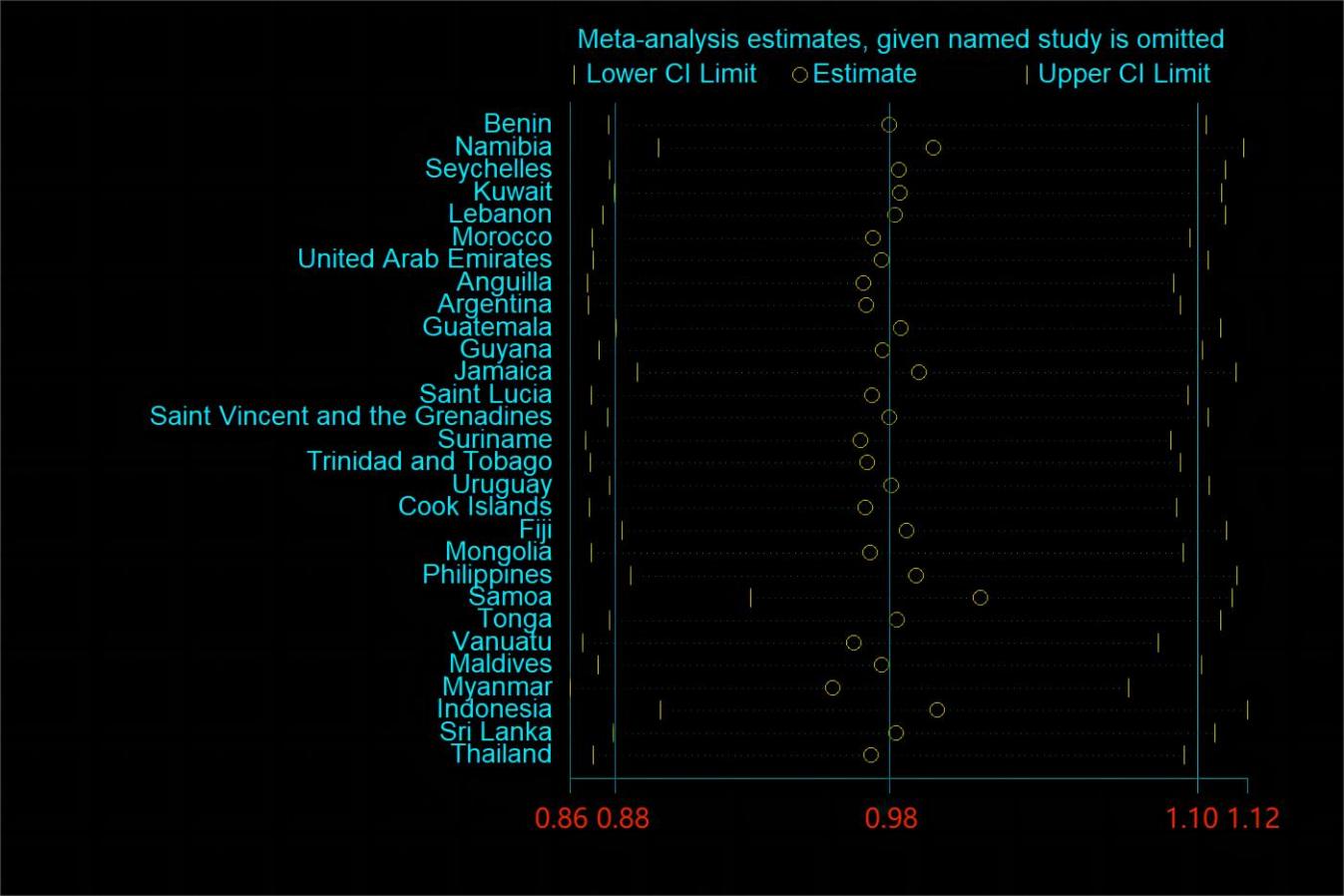


**SUPPLEMENTARY FIGURE 4**

The plot of sensitivity analysis. The circles and dashed lines represent the pooled OR and 95% CI of removing given named country. Three vertical lines represent all countries pooled OR and 95% CI.

**SUPPLEMENTARY TABLE 1**

Trends in prevalence and difference of anxiety in 29 countries

|  |  | **Prevalence (95% CI)** | **Difference (95% CI)** | **OR (95% CI)** |
| --- | --- | --- | --- | --- |
| Benin | 2009 | 14.98 (12.37-17.60) | 1.19 (-3.20-5.59) | 0.99 (0.69-1.41) |
|  | 2016 | 13.79 (10.16-17.42) |  |  |
| Namibia | 2004 | 19.78 (17.83-21.73) | 6.51 (3.88-9.14) | **0.62 (0.51-0.76)** |
|  | 2013 | 13.28 (11.45-15.10) |  |  |
| Seychelles | 2007 | 12.11 (11.55-12.67) | 1.16 (-0.68-3.00) | 0.92 (0.77-1.09) |
|  | 2015 | 10.95 (9.36-12.53) |  |  |
| Kuwait | 2011 | 19.36 (16.80-21.91) | 1.04 (-3.18-5.27) | 0.89 (0.70-1.13) |
|  | 2015 | 18.31 (14.80-21.82) |  |  |
| Lebanon | 2005 | 12.76 (11.39-14.13) | 0.85 (-0.88-2.58) | 0.96 (0.82-1.11) |
|  | 2011 | 10.38 (8.33-12.43) |  |  |
|  | 2017 | 11.91 (10.78-13.04) |  |  |
| Morocco | 2006 | 13.64 (11.34-15.93) | -0.63 (-3.15-1.89) | 1.17 (0.94-1.46) |
|  | 2010 | 14.56 (12.08-17.04) |  |  |
|  | 2016 | 14.26 (12.96-15.56) |  |  |
| United Arab Emirates | 2005 | 12.76 (11.73-13.78) | -1.49 (-3.33-0.35) | 1.08 (0.93-1.25) |
|  | 2010 | 15.35 (13.45-17.24) |  |  |
|  | 2016 | 14.25 (12.67-15.82) |  |  |
| Anguilla | 2009 | 7.19 (5.25-9.12) | -2.21 (-4.98-0.57) | 1.36 (0.96-1.92) |
|  | 2016 | 9.39 (6.89-11.90) |  |  |
| Argentina | 2007 | 10.73 (8.44-13.02) | -1.71 (-4.00-0.57) | 1.25 (0.99-1.59) |
|  | 2012 | 8.39 (7.40-9.39) |  |  |
|  | 2018 | 12.44 (11.76-13.13) |  |  |
| Guatemala | 2009 | 7.11 (6.36-7.86) | 0.65 (-0.92-2.22) | 0.88 (0.67-1.15) |
|  | 2015 | 6.46 (5.07-7.85) |  |  |
| Guyana | 2004 | 12.90 (10.51-15.29) | -0.90 (-3.55-1.75) | 1.07 (0.86-1.33) |
|  | 2010 | 13.80 (12.37-15.23) |  |  |
| Jamaica | 2010 | 18.48 (13.68-23.28) | 7.30 (2.44-12.17) | **0.68 (0.48-0.97)** |
|  | 2017 | 11.18 (9.25-13.10) |  |  |
| Saint Lucia | 2007 | 10.76 (9.18-12.33) | -2.52 (-5.04-0.00) | 1.19 (0.95-1.48) |
|  | 2018 | 13.28 (11.26-15.29) |  |  |
| Saint Vincent and the Grenadines | 2007 | 12.96 (10.73-15.20) | -1.47 (-4.65-1.72) | 0.99 (0.73-1.34) |
|  | 2018 | 14.43 (12.08-16.78) |  |  |
| Suriname | 2009 | 9.12 (7.27-10.97) | -2.88 (-5.52--0.24) | **1.36 (1.02-1.81)** |
|  | 2016 | 12.00 (9.90-14.10) |  |  |
| Trinidad and Tobago | 2007 | 9.55 (7.81-11.29) | -2.22 (-4.97-0.53) | 1.25 (0.95-1.65) |
|  | 2011 | 6.98 (5.26-8.70) |  |  |
|  | 2017 | 11.77 (9.60-13.94) |  |  |
| Uruguay | 2006 | 5.24 (4.05-6.44) | -0.21 (-1.74-1.32) | 0.97 (0.70-1.34) |
|  | 2012 | 5.46 (4.45-6.46) |  |  |
| Cook Islands | 2011 | 10.42 (8.34-12.50) | -2.93 (-6.81-0.95) | 1.32 (0.94-1.83) |
|  | 2015 | 13.35 (9.47-17.23) |  |  |
| Fiji | 2010 | 14.60 (10.79-18.41) | 2.12 (-2.19-6.42) | 0.82 (0.63-1.08) |
|  | 2016 | 12.48 (10.05-14.91) |  |  |
| Mongolia | 2010 | 4.36 (3.58-5.13) | -0.77 (-1.95-0.42) | 1.23 (0.93-1.62) |
|  | 2013 | 5.12 (4.19-6.05) |  |  |
| Philippines | 2003 | 13.74 (11.81-15.67) | 3.36 (1.17-5.55) | **0.76 (0.62-0.93)** |
|  | 2007 | 12.40 (11.05-13.74) |  |  |
|  | 2011 | 10.50 (9.24-11.76) |  |  |
|  | 2015 | 10.39 (9.31-11.46) |  |  |
| Samoa | 2011 | 27.44 (23.87-31.02) | 18.35 (14.57-22.13) | **0.30 (0.23-0.39)** |
|  | 2017 | 9.09 (7.41-10.78) |  |  |
| Tonga | 2010 | 15.54 (13.73-17.35) | 1.96 (-0.53-4.45) | 0.93 (0.76-1.14) |
|  | 2017 | 13.58 (11.84-15.32) |  |  |
| Vanuatu | 2011 | 4.73 (2.21-7.25) | -2.56 (-5.42-0.29) | **1.74 (1.06-2.85)** |
|  | 2016 | 7.29 (5.62-8.96) |  |  |
| Maldives | 2009 | 13.19 (11.13-15.25) | -0.82 (-3.59-1.94) | 1.08 (0.87-1.34) |
|  | 2014 | 14.01 (12.13-15.89) |  |  |
| Myanmar | 2007 | 1.75 (1.05-2.45) | -1.84 (-2.88--0.81) | **2.23 (1.43-3.48**) |
|  | 2016 | 3.59 (2.79-4.39) |  |  |
| Indonesia | 2007 | 7.47 (6.00-8.93) | 3.16 (1.57-4.74) | **0.58 (0.45-0.75)** |
|  | 2015 | 4.31 (3.60-5.03) |  |  |
| Sri Lanka | 2008 | 4.40 (3.57-5.22) | 0.54 (-0.81-1.89) | 0.92 (0.66-1.27) |
|  | 2016 | 3.86 (2.74-4.98) |  |  |
| Thailand | 2008 | 6.55 (5.29-7.82) | -1.64 (-4.00-0.72) | 1.23 (0.89-1.71) |
|  | 2015 | 8.19 (6.13-10.25) |  |  |
| First survey |  | 11.35 (9.57-13.12) | 0.54 (-0.53-1.61) | 0.98 (0.88-1.10) |
| Second survey |  | 10.67 (9.13-12.21) |  |  |

**SUPPLEMENTARY TABLE 2**

Trends in prevalence and difference of anxiety in girls in 29 countries

|  |  | Prevalence (95% CI) | Difference (95% CI) | OR (95% CI) |
| --- | --- | --- | --- | --- |
| Benin | 2009 | 13.45 (10.37-16.53) | -2.52 (-7.12-2.08) | 1.41 (0.95-2.08) |
|  | 2016 | 15.97 (12.34-19.60) |  |  |
| Namibia | 2004 | 20.85 (18.81-22.88) | 7.16 (4.07-10.24) | **0.61 (0.49-0.77)** |
|  | 2013 | 13.69 (11.30-16.08) |  |  |
| Seychelles | 2007 | 12.63 (11.74-13.52) | 0.09 (-2.57-2.76) | 0.98 (0.79-1.21) |
|  | 2015 | 12.54 (10.29-14.78) |  |  |
| Kuwait | 2011 | 23.73 (21.32-26.13) | 0.46 (-3.75-4.68) | 0.90 (0.72-1.12) |
|  | 2015 | 23.26 (20.01-26.52) |  |  |
| Lebanon | 2005 | 16.64 (14.77-18.51) | 2.32 (-0.14-4.78) | 0.86 (0.71-1.03) |
|  | 2011 | 12.65 (10.18-15.13) |  |  |
|  | 2017 | 14.32 (12.62-16.02) |  |  |
| Morocco | 2006 | 16.43 (13.10-19.77) | 0.40 (-3.37-4.16) | 1.09 (0.80-1.47) |
|  | 2010 | 18.28 (15.48-21.07) |  |  |
|  | 2016 | 16.04 (13.93-18.14) |  |  |
| United Arab Emirates | 2005 | 16.21 (15.16-17.26) | -2.19 (-4.81-0.43) | 1.07 (0.91-1.27) |
|  | 2010 | 17.81 (16.08-19.55) |  |  |
|  | 2016 | 18.40 (16.04-20.75) |  |  |
| Anguilla | 2009 | 9.07 (9.07-9.07) | -2.39 (-6.48-1.70) | 1.29 (0.84-1.98) |
|  | 2016 | 11.45 (7.36-15.55) |  |  |
| Argentina | 2007 | 13.31 (10.23-16.38) | -4.08 (-7.19--0.97) | **1.41 (1.09-1.83)** |
|  | 2012 | 11.33 (9.95-12.72) |  |  |
|  | 2018 | 17.38 (16.33-18.44) |  |  |
| Guatemala | 2009 | 9.02 (7.75-10.28) | 0.91 (-1.48-3.30) | 0.85 (0.63-1.15) |
|  | 2015 | 8.10 (6.03-10.18) |  |  |
| Guyana | 2004 | 14.92 (11.89-17.94) | -0.03 (-3.61-3.55) | 0.98 (0.73-1.32) |
|  | 2010 | 14.94 (12.72-17.17) |  |  |
| Jamaica | 2010 | 23.11 (15.67-30.55) | 9.97 (2.27-17.68) | **0.60 (0.38-0.95)** |
|  | 2017 | 13.14 (9.75-16.53) |  |  |
| Saint Lucia | 2007 | 13.43 (10.79-16.06) | -2.29 (-6.63-2.06) | 1.10 (0.79-1.54) |
|  | 2018 | 15.71 (12.18-19.25) |  |  |
| Saint Vincent and the Grenadines | 2007 | 14.41 (10.88-17.94) | -4.58 (-9.56-0.40) | 1.17 (0.80-1.72) |
|  | 2018 | 18.99 (15.38-22.61) |  |  |
| Suriname | 2009 | 10.14 (6.44-13.84) | -4.66 (-9.00--0.33) | 1.54 (0.99-2.39) |
|  | 2016 | 14.81 (12.28-17.33) |  |  |
| Trinidad and Tobago | 2007 | 13.21 (10.20-16.23) | -1.57 (-5.66-2.53) | 1.17 (0.84-1.65) |
|  | 2011 | 8.83 (6.30-11.37) |  |  |
|  | 2017 | 14.78 (11.99-17.56) |  |  |
| Uruguay | 2006 | 6.58 (4.93-8.23) | -1.25 (-3.65-1.14) | 1.11 (0.76-1.62) |
|  | 2012 | 7.83 (6.03-9.64) |  |  |
| Cook Islands | 2011 | 13.85 (13.85-13.85) | -2.56 (-8.60-3.49) | 1.19 (0.79-1.80) |
|  | 2015 | 16.41 (10.36-22.46) |  |  |
| Fiji | 2010 | 13.78 (11.07-16.50) | 1.75 (-2.15-5.65) | 0.78 (0.57-1.08) |
|  | 2016 | 12.04 (9.00-15.08) |  |  |
| Mongolia | 2010 | 4.69 (3.59-5.80) | -0.32 (-1.88-1.25) | 1.12 (0.78-1.60) |
|  | 2013 | 5.01 (3.86-6.16) |  |  |
| Philippines | 2003 | 16.01 (13.95-18.06) | 3.53 (0.92-6.13) | **0.74 (0.59-0.92)** |
|  | 2007 | 13.72 (10.99-16.45) |  |  |
|  | 2011 | 11.35 (9.34-13.36) |  |  |
|  | 2015 | 12.48 (10.84-14.12) |  |  |
| Samoa | 2011 | 27.86 (22.60-33.12) | 18.68 (13.36-24.00) | **0.30 (0.21-0.43)** |
|  | 2017 | 9.18 (7.33-11.03) |  |  |
| Tonga | 2010 | 15.85 (13.81-17.88) | 1.76 (-1.26-4.79) | 0.96 (0.75-1.23) |
|  | 2017 | 14.08 (11.82-16.35) |  |  |
| Vanuatu | 2011 | 4.76 (2.33-7.19) | -2.59 (-5.86-0.69) | 1.49 (0.86-2.58) |
|  | 2016 | 7.34 (5.00-9.68) |  |  |
| Maldives | 2009 | 16.20 (13.52-18.87) | -1.42 (-5.62-2.77) | 1.11 (0.83-1.49) |
|  | 2014 | 17.62 (14.34-20.91) |  |  |
| Myanmar | 2007 | 1.42 (0.57-2.27) | -2.25 (-3.72--0.78) | **2.92 (1.50-5.71)** |
|  | 2016 | 3.67 (2.42-4.91) |  |  |
| Indonesia | 2007 | 7.62 (5.64-9.61) | 3.65 (1.55-5.74) | **0.52 (0.37-0.73)** |
|  | 2015 | 3.97 (3.13-4.81) |  |  |
| Sri Lanka | 2008 | 4.44 (3.10-5.78) | 0.48 (-1.02-1.99) | 0.91 (0.61-1.35) |
|  | 2016 | 3.96 (3.16-4.75) |  |  |
| Thailand | 2008 | 6.52 (5.19-7.84) | -1.38 (-4.13-1.36) | 1.16 (0.79-1.72) |
|  | 2015 | 7.90 (5.42-10.37) |  |  |
| First survey |  | 12.91 (10.64-15.19) | 0.32 (-0.89-1.53) | 0.97 (0.87-1.10) |
| Second survey |  | 12.48 (10.40-14.61) |  |  |

**SUPPLEMENTARY TABLE 3**

Trends in prevalence and difference of anxiety in boys in 29 countries

|  |  | Prevalence (95% CI) | Difference (95% CI) | OR (95% CI) |
| --- | --- | --- | --- | --- |
| Benin | 2009 | 15.59 (12.25-18.92) | 3.15 (-2.31-8.62) | 0.80 (0.51-1.27) |
|  | 2016 | 12.43 (7.85-17.02) |  |  |
| Namibia | 2004 | 18.82 (15.93-21.71) | 6.09 (2.19-9.99) | **0.64 (0.47-0.86)** |
|  | 2013 | 12.73 (9.99-15.47) |  |  |
| Seychelles | 2007 | 11.61 (10.91-12.32) | 2.24 (0.07-4.42) | 0.81 (0.63-1.06) |
|  | 2015 | 9.37 (7.39-11.34) |  |  |
| Kuwait | 2011 | 14.99 (10.55-19.43) | 1.93 (-3.47-7.33) | 0.89 (0.60-1.31) |
|  | 2015 | 13.06 (9.45-16.67) |  |  |
| Lebanon | 2005 | 8.41 (7.07-9.75) | -0.82 (-3.24-1.60) | 1.15 (0.86-1.53) |
|  | 2011 | 7.77 (5.27-10.27) |  |  |
|  | 2017 | 9.23 (7.10-11.35) |  |  |
| Morocco | 2006 | 11.00 (8.26-13.73) | -1.73 (-4.66-1.20) | 1.26 (0.96-1.66) |
|  | 2010 | 11.51 (8.63-14.38) |  |  |
|  | 2016 | 12.73 (11.26-14.20) |  |  |
| United Arab Emirates | 2005 | 9.04 (8.26-9.83) | -0.62 (-2.41-1.18) | 1.08 (0.89-1.32) |
|  | 2010 | 11.47 (9.66-13.28) |  |  |
|  | 2016 | 9.66 (8.16-11.17) |  |  |
| Anguilla | 2009 | 5.41 (5.41-5.41) | -2.12 (-5.26-1.01) | 1.49 (0.91-2.45) |
|  | 2016 | 7.53 (4.39-10.67) |  |  |
| Argentina | 2007 | 7.57 (5.31-9.84) | 0.37 (-1.88-2.62) | 0.96 (0.69-1.34) |
|  | 2012 | 5.25 (4.26-6.23) |  |  |
|  | 2018 | 7.21 (6.59-7.82) |  |  |
| Guatemala | 2009 | 5.32 (4.12-6.52) | 0.19 (-1.63-2.00) | 0.92 (0.57-1.49) |
|  | 2015 | 5.13 (3.79-6.48) |  |  |
| Guyana | 2004 | 10.58 (5.97-15.19) | -1.97 (-7.27-3.33) | 1.21 (0.74-2.00) |
|  | 2010 | 12.55 (9.43-15.67) |  |  |
| Jamaica | 2010 | 14.17 (10.47-17.88) | 5.22 (1.10-9.35) | 0.84 (0.56-1.25) |
|  | 2017 | 8.95 (6.63-11.27) |  |  |
| Saint Lucia | 2007 | 7.39 (4.41-10.37) | -3.67 (-7.60-0.26) | 1.36 (0.83-2.22) |
|  | 2018 | 11.06 (8.38-13.74) |  |  |
| Saint Vincent and the Grenadines | 2007 | 11.19 (8.09-14.29) | 2.14 (-1.80-6.07) | 0.73 (0.44-1.22) |
|  | 2018 | 9.05 (6.50-11.59) |  |  |
| Suriname | 2009 | 8.04 (3.73-12.35) | -0.99 (-5.62-3.65) | 1.11 (0.60-2.06) |
|  | 2016 | 9.02 (6.79-11.26) |  |  |
| Trinidad and Tobago | 2007 | 5.91 (4.43-7.39) | -2.73 (-5.47-0.01) | 1.43 (0.96-2.12) |
|  | 2011 | 5.08 (3.36-6.80) |  |  |
|  | 2017 | 8.65 (6.30-10.99) |  |  |
| Uruguay | 2006 | 3.58 (2.28-4.89) | 0.86 (-0.65-2.37) | 0.69 (0.42-1.11) |
|  | 2012 | 2.72 (1.91-3.53) |  |  |
| Cook Islands | 2011 | 7.31 (7.31-7.31) | -2.62 (-7.52-2.28) | 1.55 (0.86-2.79) |
|  | 2015 | 9.94 (4.99-14.88) |  |  |
| Fiji | 2010 | 15.47 (9.87-21.07) | 1.62 (-4.78-8.02) | 0.87 (0.58-1.32) |
|  | 2016 | 13.85 (10.13-17.57) |  |  |
| Mongolia | 2010 | 3.93 (2.89-4.98) | -1.32 (-2.72-0.09) | 1.35 (0.96-1.91) |
|  | 2013 | 5.25 (4.26-6.23) |  |  |
| Philippines | 2003 | 10.40 (7.90-12.91) | 2.28 (-0.57-5.12) | 0.80 (0.58-1.12) |
|  | 2007 | 10.87 (8.95-12.78) |  |  |
|  | 2011 | 9.60 (8.06-11.14) |  |  |
|  | 2015 | 8.13 (6.72-9.53) |  |  |
| Samoa | 2011 | 27.14 (23.53-30.75) | 17.84 (13.60-22.09) | **0.30 (0.21-0.42)** |
|  | 2017 | 9.29 (6.85-11.73) |  |  |
| Tonga | 2010 | 15.23 (12.49-17.97) | 2.23 (-1.38-5.85) | 0.88 (0.65-1.18) |
|  | 2017 | 13.00 (10.60-15.39) |  |  |
| Vanuatu | 2011 | 4.98 (1.66-8.29) | -2.25 (-6.25-1.75) | **2.28 (1.09-4.73)** |
|  | 2016 | 7.23 (4.64-9.81) |  |  |
| Maldives | 2009 | 10.18 (7.30-13.06) | -0.43 (-4.24-3.37) | 1.04 (0.69-1.55) |
|  | 2014 | 10.61 (8.07-13.15) |  |  |
| Myanmar | 2007 | 2.08 (1.13-3.03) | -1.44 (-2.66--0.22) | **1.70 (1.03-2.83)** |
|  | 2016 | 3.53 (2.71-4.34) |  |  |
| Indonesia | 2007 | 7.25 (5.45-9.05) | 2.55 (0.60-4.51) | **0.65 (0.47-0.89)** |
|  | 2015 | 4.69 (3.80-5.59) |  |  |
| Sri Lanka | 2008 | 4.46 (2.87-6.05) | 0.69 (-1.64-3.02) | 0.93 (0.52-1.66) |
|  | 2016 | 3.77 (2.04-5.50) |  |  |
| Thailand | 2008 | 6.57 (5.02-8.12) | -1.91 (-4.97-1.15) | 1.29 (0.84-1.98) |
|  | 2015 | 8.48 (5.76-11.20) |  |  |
| First survey |  | 9.52 (7.96-11.09) | 0.68 (-0.41-1.76) | 0.97 (0.85-1.11) |
| Second survey |  | 8.70 (7.53-9.86) |  |  |
